# Iterative and prolonged remission in metastatic breast cancer using pegylated irinotecan: a case report

**DOI:** 10.1186/1752-1947-9-5

**Published:** 2015-02-12

**Authors:** Mounia Amzerin, Maha Mokrim, Hassan Errihani, Martine J Piccart

**Affiliations:** 3 Department of Medical Oncology, National Institute of Oncology, Université Mohammed V de Rabat, Avenue Allal El Fassi, 10100 Rabat, Morocco; 4 Department of Medical Oncology, Jules Bordet Institute, Université Libre de Bruxelles, Boulevard de Waterloo 125, B-1000 Brussels, Belgium

**Keywords:** NKTR-102, Pegylated irinotecan, Metastatic breast cancer

## Abstract

**Introduction:**

Pegylated irinotecan NKTR-102 is a topoisomerase I inhibitor-polymer conjugate. This new formulation of irinotecan has been evaluated in a phase II clinical trial and is showing remarkable activity. To the best of our knowledge, this is the first case report of an impressive iterative response to pegylated irinotecan NKTR-102 in metastatic breast cancer.

**Case presentation:**

We report the case of a 49-year-old Caucasian woman diagnosed with metastatic luminal A breast cancer with initial bone followed by liver and bone marrow metastases, treated with three lines of hormonal therapy, targeted therapy and six lines of chemotherapy. She showed no major response to conventional treatment, whereas, the tumor shrinkage under pegylated irinotecan NKTR-102 was impressive, durable and iterative.

**Conclusions:**

Reintroduction of an active drug is a valid approach as illustrated by our case. The results of the current phase III trials of pegylated irinotecan NKTR-102 are eagerly awaited.

## Introduction

Metastatic breast cancer is an incurable disease. Systemic therapy aims to prolong disease control while preserving good quality of life. During past years, many drugs have been developed and have proven efficacy, leading to some prolongation of overall survival. The active search for new anticancer drugs continues while new formulations of the existing ones are also being pursued. Irinotecan, a topoisomerase I inhibitor and a major cytotoxic drug for some tumor types, has limited activity in breast cancer. Thus, it has been reformulated by pegylation, in order to reach a durable high concentration in tumor tissue, with the hope that its antitumor spectrum activity will be broadened. This new formulation, NKTR-102, has shown promising activity in phase II studies and it is currently being evaluated in phase III clinical trials [1]. We report the case of a patient, enrolled in a clinical trial with pegylated irinotecan, who showed an impressive and prolonged response even after the drug’s reintroduction.

## Case presentation

**Figure 1:**
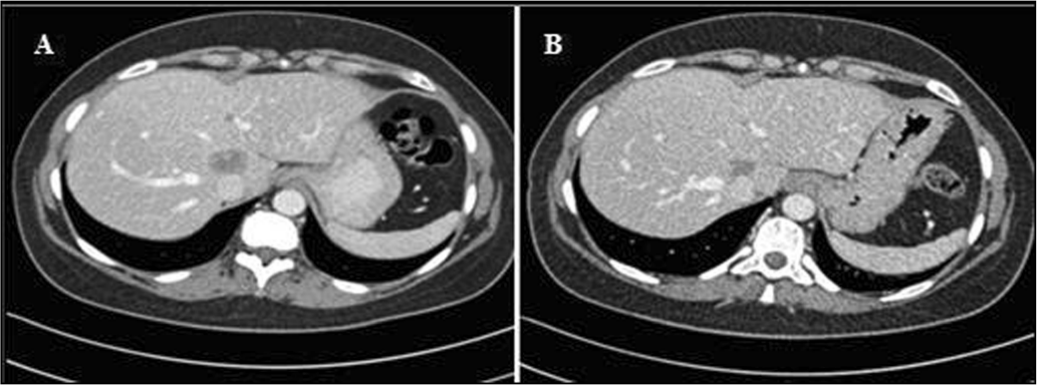
**Tumor response after the first three cycles of NKTR-102. A**: Baseline abdominal computed tomography scan showing liver metastasis. **B**: Abdominal computed tomography scan showing a partial response after three cycles of pegylated irinotecan NKTR-102.

**Figure 2:**
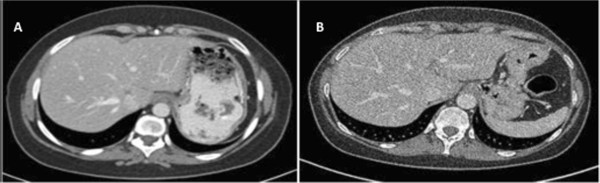
**Tumor response after NKTR-102 discontinuation. A**: Partial response at the 12th cycle of pegylated irinotecan NKTR-102. **B**: Abdominal computed tomography scan four months after the last course of pegylated irinotecan NKTR-102, showing additional response.

A 49-year-old premenopausal Caucasian woman was diagnosed in June 2006 with stage IV luminal A breast cancer and bone metastases. She had a medical history of smoking during fifteen years, grade A esophagitis (Savary and Miller classification) and an iodine allergy. She had no family history of cancer. Since luminal A breast cancer is hormone-sensitive and she was asymptomatic, she received a first-line hormonal therapy consisting of tamoxifen (20mg, once daily) combined with a luteinizing hormone-releasing hormone agonist every three months, resulting in a partial response after six months. She underwent a lumpectomy, sentinel node biopsy and concomitant surgical castration. Tamoxifen was continued after her surgery. The disease progressed three months later with the emergence of liver metastases. She received anastrozole (1mg, once daily) + gefitinib (250mg, per day) in a clinical trial, with disease progression after three months. Her treatment was then switched to chemotherapy associating cyclophosphamide (600mg/m2) and epirubicin (75mg/m2, every three weeks), which induced a partial response after three and a half months (six cycles). Exemestane (25mg, daily) was given as a maintenance treatment until the disease progressed 15 months later; her CA 15-3 level increased, new liver metastases appeared and an osteo-medullary infiltration was diagnosed.She was treated with docetaxel (100mg/m2, every three weeks) and showed a good response after four and half months (seven cycles). After a short treatment break of two months, the disease progressed once more; the CA 15-3 level was 102UI/mL. She was enrolled in a clinical trial and received pegylated irinotecan NKTR-102 at 145mg/m2 every two weeks. After one and a half months, the response assessment showed a partial radiological response of the liver metastases according to RECIST (Figure 1), and the CA 15-3 marker level had decreased to the normal range. The response was maintained for five and a half months (12 cycles; Figure 2A) but the treatment had to be stopped due to uninterrupted grade 3 diarrhea (NCI-CTC scale, version 4.0). Four months later, further liver metastases shrinkage was noted even in the absence of treatment (Figure 2B). She enjoyed a period of disease stability with no treatment for 18 months.

After this period, liver metastases were found to have progressed and pegylated irinotecan was reintroduced. This, once again, induced a 50% partial radiological response and a decrease of the CA 15-3 level from 896 to 173UI/mL after three cycles. Her liver disease continued to respond for an additional eight months, until it progressed in September 2012. The main toxicities caused by NKTR-102 were alopecia, diarrhea, asthenia and anorexia, all grade I (NCI-CTC scale, version 4.0). There was no cumulative toxicity. She then received capecitabine (1250mg/m2, twice daily from day one to 14, every three weeks), but liver metastases progression was documented after only four months. She subsequently received eribulin (1.23mg/m2, every three weeks) as the fifth line of chemotherapy. Her liver disease progressed once again in the fifth month of treatment. She underwent a biopsy of a liver metastasis which revealed a non-hormone-sensitive Her2+ tumor clone. She received a sixth line of chemotherapy consisting of weekly paclitaxel combined with trastuzumab and zoledronate. Her liver disease was in complete response while her bone metastases remained stable. Eight years after the initial diagnosis, she is still surviving and is currently under a maintenance therapy consisting of letrozole, trastuzumab and denosumab for her bone lesions.

## Discussion

Irinotecan is a topoisomerase I inhibitor approved for the treatment of metastatic breast cancer in Japan [2]. An objective response rate (ORR) of 23% was obtained in an American phase II study which was comparable to Japanese literature data [3, 4]. In European metastatic breast cancer patients, irinotecan has a reputation of having limited activity in advanced breast cancer [2, 5]. That said, clinical trials with irinotecan in metastatic breast cancer are limited [6].

Pegylated irinotecan NKTR-102 is a long acting topoisomerase I inhibitor [7]. It was obtained by conjugating irinotecan to a four-arm polyethylene glycol [8]. This interesting new formulation achieves an elimination half-life of the active metabolite SN-38 of 50 days, compared to two days with irinotecan, while the peak concentrations are five to 10 times lower [7]. Thus, there is an uninterrupted exposure to SN-38 and less toxicity [7]. This had been illustrated by preclinical data demonstrating the superiority of pegylated irinotecan NKTR-102 to irinotecan in lung, breast and colorectal mouse xenografts models [9]. Phase I studies showed an encouraging activity in various solid tumors and recommended a dose of 145mg/m2 every 14 or 21 days for phase II clinical trials [7]. Subsequent phase II studies revealed remarkable objective response rates in taxane-refractory metastatic breast cancer (29%) and platinum-resistant or refractory ovarian cancer (20%), respectively [1, 10].

Metastatic breast cancer is a chronic disease for which the goal of systemic therapies is to achieve prolonged disease stabilization while maintaining quality of life. Our patient experienced an objective response to the successive lines of chemotherapy and endocrine therapy she received. However, the disease was progressing each time after a short period of chemotherapy. The literature provides little data regarding prolonged tumor responses under chemotherapy. Anectotal durable remissions have been observed with capecitabine, gemcitabine and vinorelbine (Table 1) [11–13]. Long remissions have mainly been reported with biological treatments [14, 15]. For our patient, the introduction of pegylated irinotecan NKTR-102 gave, for the first time, a durable response with manageable toxicity. Interestingly, disease regression continued even after the treatment was stopped. This illustrates the particularity of NKTR-102’s pharmacokinetic profile, namely, conferring longer half time and more prolonged exposure to the active drug.

**Table 1:** **Prolonged progression-free survival (PFS) anecdotally reported in metastatic breast cancer refractory to anthracyclines and taxanes**

Drug	Prolonged PFS (months) observed individually in the literature	Reference
Eribulin	NR	
Vinorelbine	12 for responding patients	Zelek *et al*. [11]
Ixabepilone	NR	
Capecitabine	12	Rogers *et al*. [12]
Gemcitabine	23	Schmid *et al*. [13]

NR: Not reported.

The durable remission for 18 months without any maintenance treatment is another interesting feature in our case. This could be partially explained by the ‘favorable’ histology of the disease, however, the other treatments provided only short periods of remission. Therefore, we suggest that pegylated irinotecan NKTR-102 played a role in this prolonged control of the disease, by some mechanism which remains to be elucidated.

After the sustainable impressive response to pegylated irinotecan NKTR-102, the decision to reintroduce it after disease progression seemed logical in view of the fact that our patient had enjoyed a good response in the one year before her liver metastases started to progress again. After an initial response and a prolonged progression-free interval, the reintroduction of an active drug in front of tumor progression is a common practice since one assumes no resistance to the drug has developed. The case of our patient confirms the validity of this approach.

Our patient belongs respectively to the ranges of 0 to 37% [16] and 14.5 to 40% [16] of patients who experience the acquisition of a Her2+ profile and the discordance in estrogen ER and progesterone PR receptors expression. A novel biopsy was very useful in this case since a completely different clone of tumor cells was identified and she could be offered a more personalized treatment option.

## Conclusions

The case of our patient illustrates the huge progress made in the management of breast cancer. Thanks to clinical research, more treatments and options are currently available. This allows patients to enjoy prolonged remissions and improved survival. The identification of a new tumor clone made in our case supports the notion that a biopsy of metastatic sites in cases of disease progression is not a luxury but a necessity. In addition, as stated above, the new design and formulation of a known drug can be an elegant way of broadening its antitumor spectrum. Pegylated irinotecan NKTR-102 is currently being evaluated in a phase III pivotal trial for metastatic breast cancer after anthracyclines, taxanes and capecitabine [17]. Results from this trial are eagerly awaited.

## Consent

Written informed consent was obtained from the patient for publication of this case report and any accompanying images. A copy of the written consent is available for review by the Editor-in-Chief of this journal.
